# Supplementary material for: Repeated exposure with short-term behavioral stress resolves pre-existing stress-induced depressive-like behavior in mice
Source: Nat Commun. 2021 Nov 18;12:6682. doi: 10.1038/s41467-021-26968-4 (PMC8602389; doi:10.1038/s41467-021-26968-4)
Supplement: Supplementary file 1 — Supplementary Information [file 41467_2021_26968_MOESM1_ESM.pdf]

SUPPLEMENTARY INFORMATION

Repeated exposure with short-term behavioral stress resolves pre-existing stress-induced depressive-like behavior in mice

Lee, E.H. et al.

***Corresponding author:** Dr. PL Han (e-mail: plhan@ewha.ac.kr, plhan@hanmail.net)

Illustrations:

Supplementary Figures 1-12

Supplementary Fig. 1

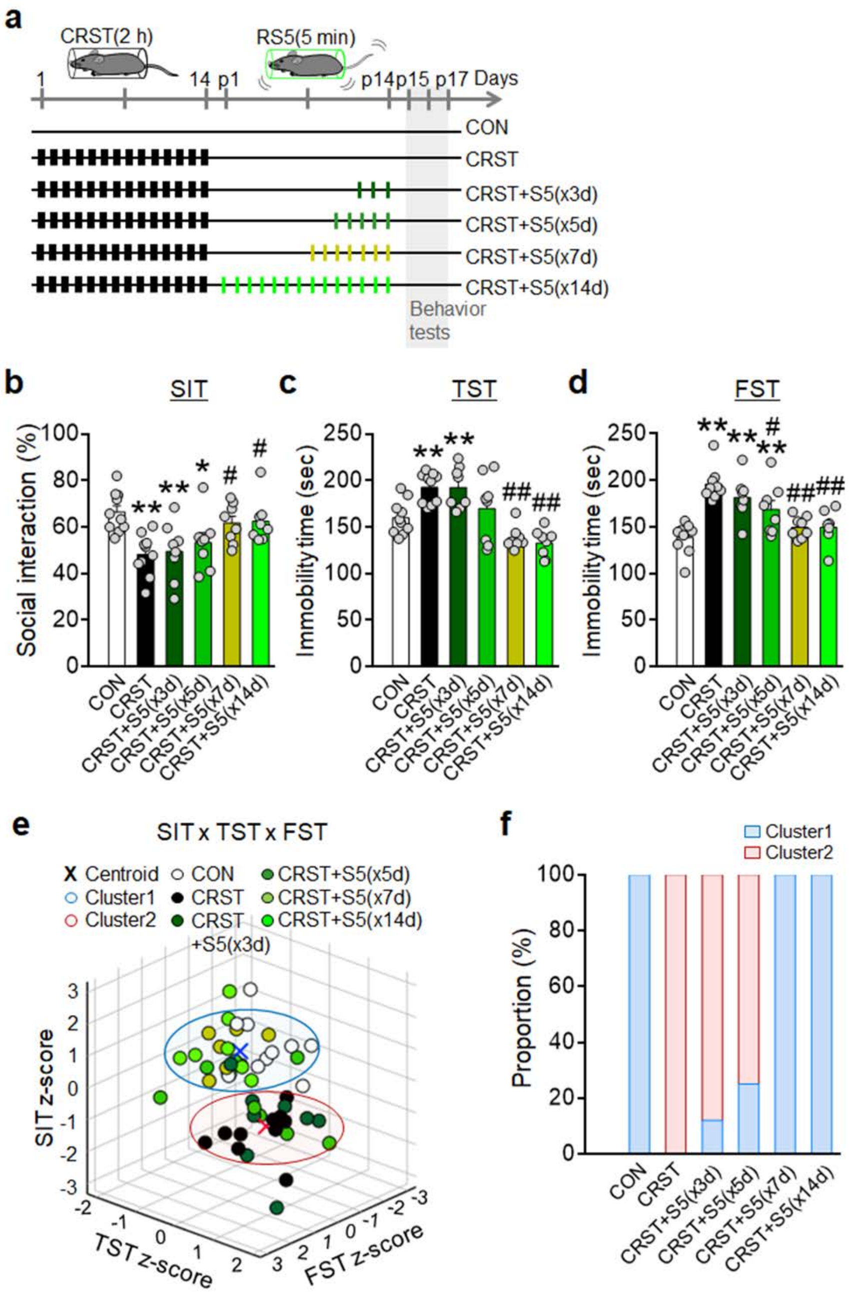

Supplementary Fig. 1 Dosing analysis of the repeatability of short-term behavioral stress in producing anti-depressive effects. a, Experimental design. CRST mice were treated with daily 5-min restraint for 3, 5, 7, or 14 days (S5x3d, S5x5d, S5x7d, and S5x14d, respectively) and then were placed in the behavioral tests as depicted. **b**–**f**, The % time of social interaction in the SIT (**b**), and immobility time in the TST (**c**) and FST (**d**) for the indicated groups. *K*-Means clustering (k = 2) of individuals in the SIT x TST x FST matrix (**e**) and the % composition of each group in the clusters (**f**) (n = 8–10 per group). Data are mean ± SEM. Gray circles represent individual data points. *, difference compared to control; #, difference compared to CRST. *, #, $p < 0.05$; **, ##, $p < 0.01$ (One-way ANOVA followed by Newman-Keuls post-hoc test). See Supplementary Data 4 for statistical details.

Supplementary Fig. 2

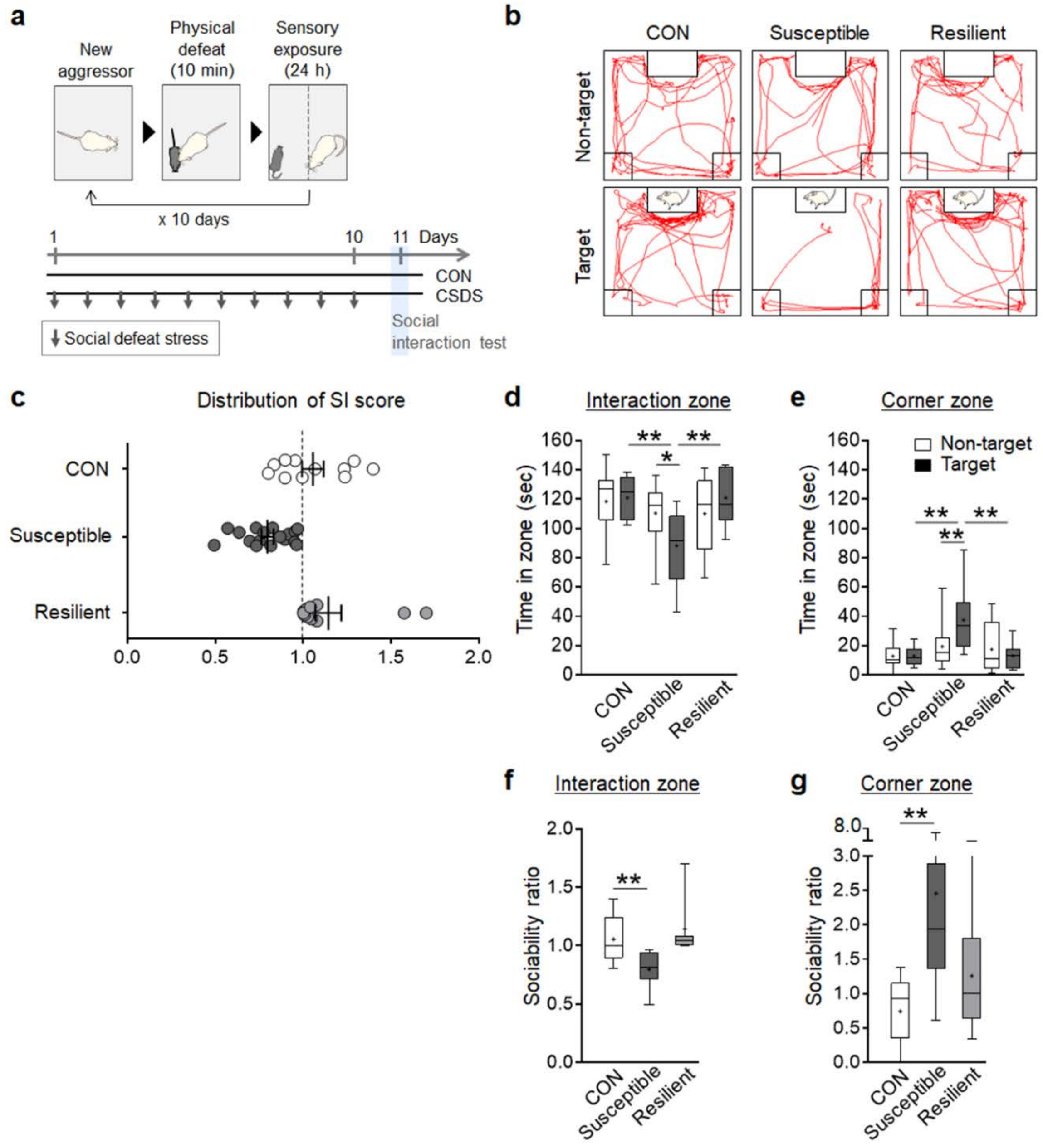

Supplementary Fig. 2 Separation of mice susceptible and resilient to social defeat stress on the basis of the social interaction test. a, Experimental design. Mice were exposed to social defeat stress for 10 days and then placed in the behavioral test to segregate susceptible and resilient mice. **b, c**, Representative tracks of susceptible and resilient mice in the open field with or without a social target in the sociability test for the indicated groups (**b**) and distribution of individual's SI scores after social defeat stress (**c**). **d–f**, Total time (sec) spent in the interaction zone (**d**) and corner zone (**e**) and the sociability ratio of susceptible and resilient mice in the interaction zone (**f**) and corner zone (**g**) for the indicated groups (*n* = 11–17 per group). Data are mean ± SEM. Gray circles represent individual data points. *, difference compared to the indicated group. *, $p < 0.05$; **, $p < 0.01$ (One-way ANOVA followed by Newman-Keuls post-hoc test). See Supplementary Data 4 for statistical details.

Supplementary Fig. 3

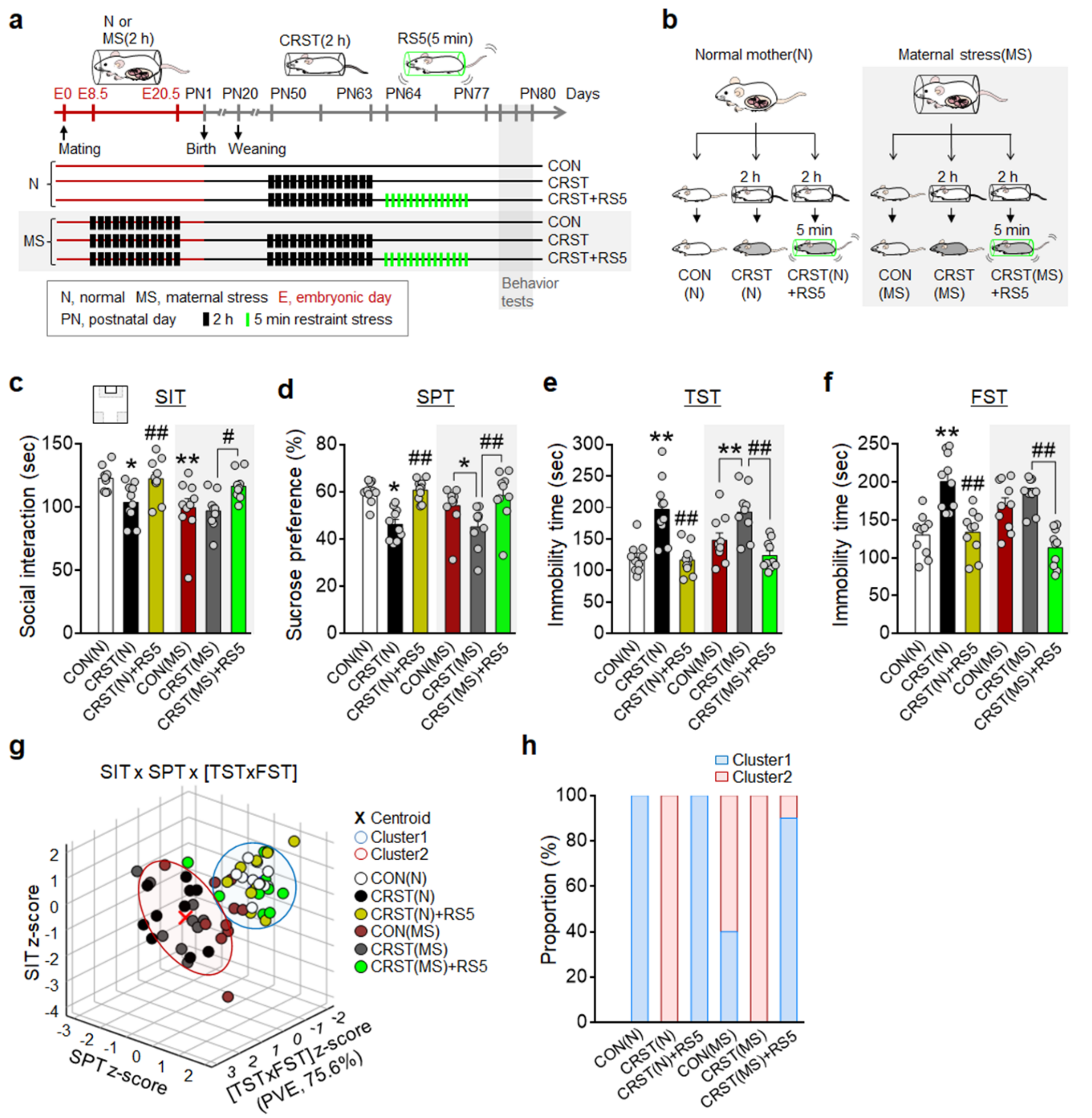

Supplementary Fig. 3 Repeated treatment with short-term behavioral stress produces anti-depressive effects in ICR mice. a,**b**, Experimental design (**a**). Pregnant ICR females were exposed to daily 2-h restraint from E8.5 until delivery (called maternal stress or MS group), and their pups (MS pups) were grown to adulthood. Pregnant normal ICR females with no stress (called normal or N group) and their pups (N pups) were prepared in parallel to use as controls. Beginning at 7 weeks of age, the MS pups and N pups were randomly allocated to treat with CRST or CRST+RS5 for 14 days as depicted (**b**). **c**–**h**, The % time of social interaction in the SIT (**c**), % sucrose preference in the SPT (**d**), and immobility time in the TST (**e**) and FST (**f**) for the indicated groups. PCA and *K*-Means cluster analysis of individual animals in the SIT x SPT x [TST x FST] matrix (**g**) and the % composition in each cluster (**h**). CRST-treated N and MS groups were shifted from the cluster containing

CRST mice to the cluster containing the control (N group) after TS5 treatment. The TST and FST components were transformed into a linear dimension by PCA (PVE; 75.6%) (n = 9–10 per group). Data are mean ± SEM. Gray circles represent individual data points. *, difference compared to control; #, difference compared to CRST. *, #, $p < 0.05$; **, ##, $p < 0.01$ (One-way ANOVA followed by Newman-Keuls post-hoc test). See Supplementary Data 4 for statistical details.

Supplementary Fig. 4

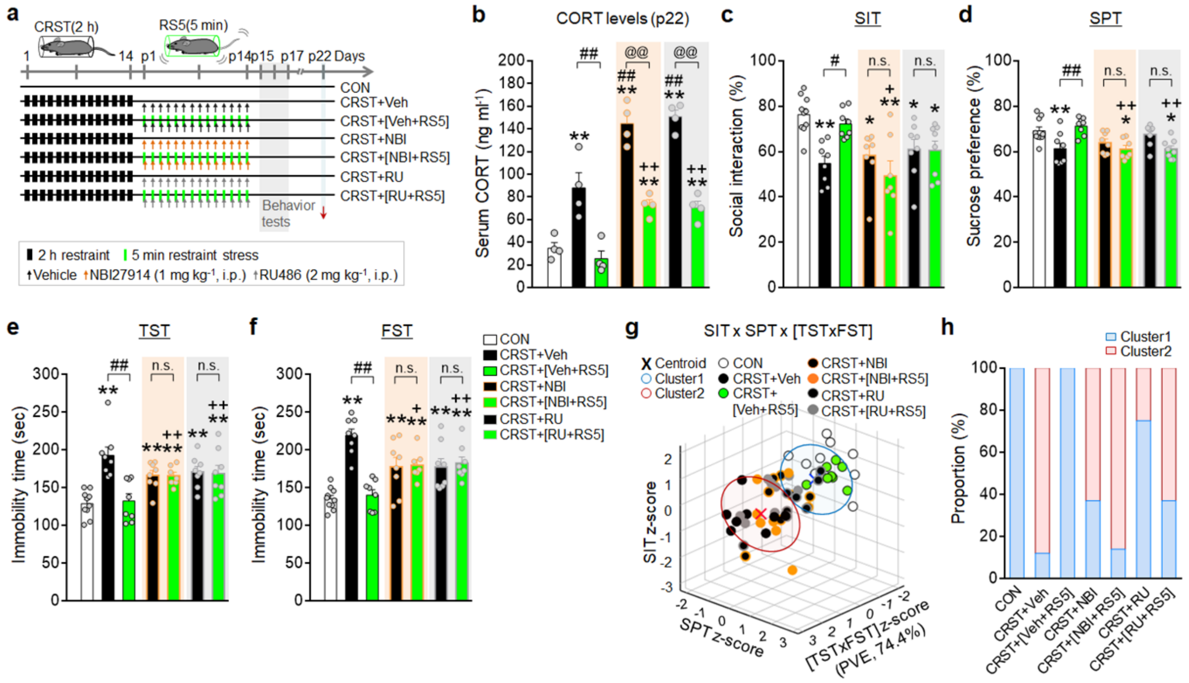

Supplementary Fig. 4 Pharmacological inhibition of the HPA axis blocks the anti-depressive effects of RS5. a, Experimental design. Mice were exposed to CRST, followed by treatment for 14 days with daily 5-min restraint 30 min after injection with NBI 27914 (1 mg kg^{-1} per injection, i.p.) or RU486 (2 mg kg^{-1} per injection, i.p.), and then placed in the behavioral tests. **b**–**h**, Basal serum CORT levels in the indicated groups (**b**) (n = 7–8 per group). The % time of social interaction in the SIT (**c**), % sucrose preference in the SPT (**d**), and immobility time in the TST (**e**) and FST (**f**) for the indicated groups. *K*-Means clustering of individuals in the SIT x SPT x [TST x FST] matrix (**g**) and the % composition of each group in the clusters (**h**). The TST and FST components were transformed into a one-dimensional variable using PCA (PVE; 74.4%) (n = 7–10 per group). Data are mean ± SEM. Gray circles represent individual data points. *, difference compared to control; #, difference compared to CRST; +, difference compared to CRST+RS5; @, difference compared to the indicated group. *, #, +, @, $p < 0.05$; **, ##, ++, @@, $p < 0.01$; n.s., not significant (One-way ANOVA followed by Newman-Keuls post-hoc test). See Supplementary Data 4 for statistical details.

Supplementary Fig. 5

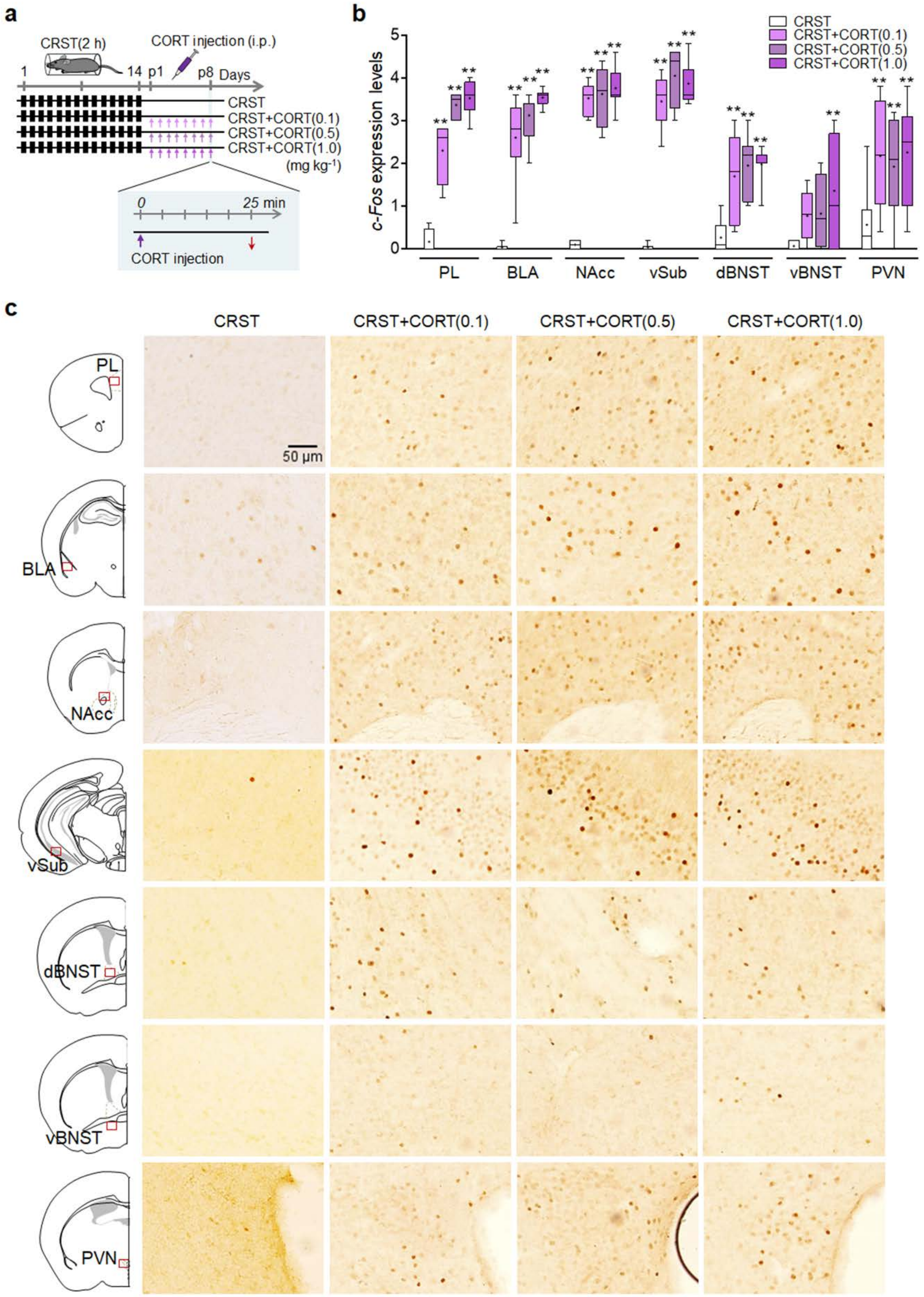

Supplementary Fig. 5 Low-dose CORT treatment activates the brain regions regulating stress coping in CRST mice. a, Experimental design. Mice were

subjected to CRST, followed by CORT injection (purple arrows) at 0.1, 0.5, or 1.0 mg kg^{-1} per day for 7 days and then sacrificed 25 min after an additional CORT injection on post-stress day 8. Red arrow, time point for tissue prep. **b**, *c-Fos* expression levels induced by CORT injection in the PL, BLA, NAcc, vSub, dBNST, vBNST, and PVN of the CRST control, CRST+CORT (0.1 mg kg^{-1}), CRST+CORT (0.5 mg kg^{-1}), and CRST+CORT (1.0 mg kg^{-1}) groups (n = 4–6 per group). **c**, Photomicrographs showing *c-Fos* expression in the PL, BLA, NAcc, vSub, dBNST, vBNST, and PVN (red boxes) for the indicated groups. The details are shown in Supplementary Table 1. Data are mean ± SEM. Gray circles represent individual data points. *, **, difference compared to control. *, $p < 0.05$; **, $p < 0.01$ (One-way ANOVA followed by Newman-Keuls post-hoc test). See Supplementary Data 4 for statistical details.

Supplementary Fig. 6

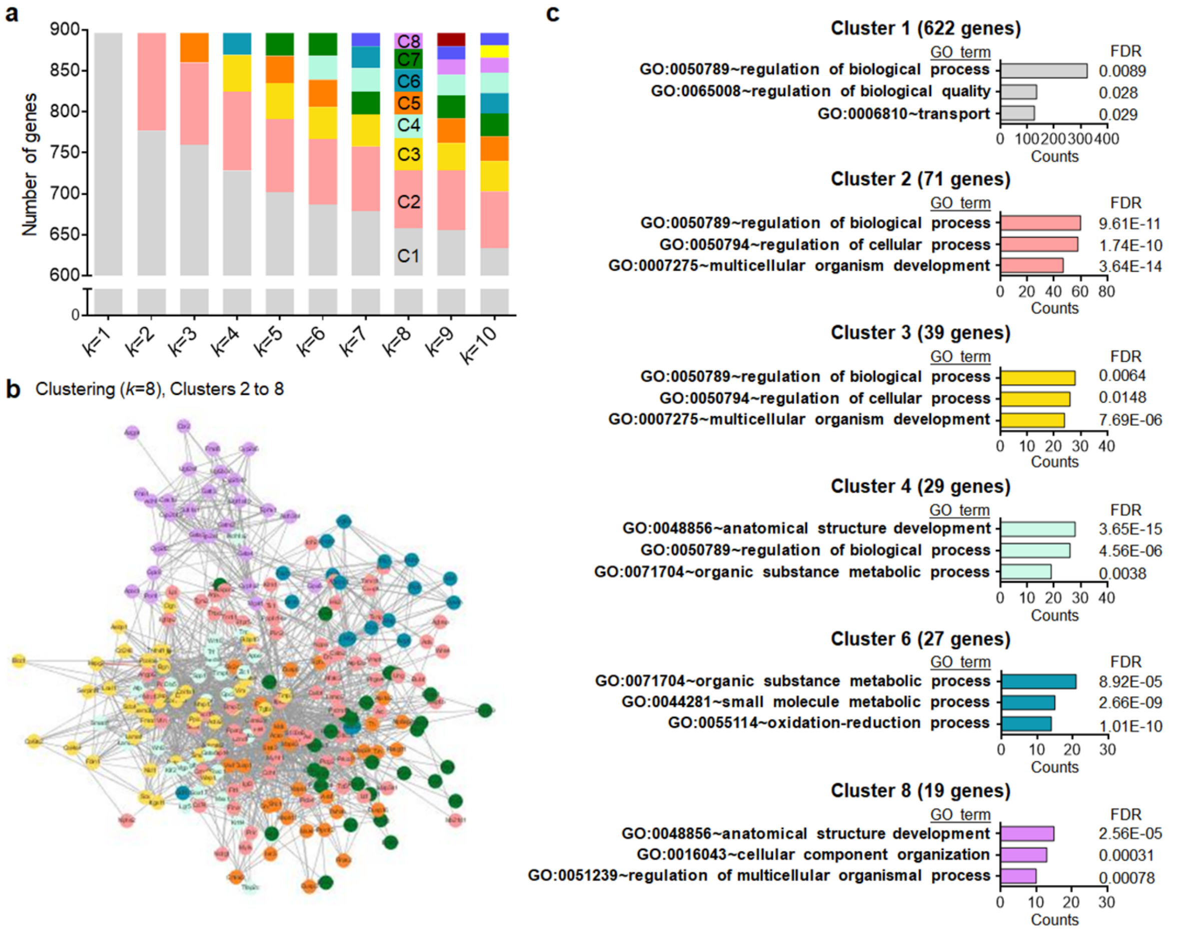

Supplementary Fig. 6 Analysis of gene expression profiles and protein–protein interaction networks in the PL changed by CRST and RS5 treatment. a, Serial *K*-Means clustering was used to group the 860 genes identified into featured clusters. Adding *k* +1 value increasingly stepped up a new cluster, and the members in a newly formed cluster were mostly supplied from the largest cluster. The cognate inter-clusters are marked with the same color code. C, cluster. Concerning the classification with *k* = 8, cluster 1 (gray) contained 622 genes; cluster 2 (scarlet) contained 71 genes; cluster 3 (yellow) contained 39 genes; cluster 4 (light green) contained 29 genes; cluster 5 (orange) contained 28 genes; cluster 6 (blue) contained 27 genes; cluster 7 (green) contained 24 genes; and cluster 8 (violet) contained 19 genes. **b**, Functional protein–protein interaction (PPI) networks are constructed with the 237 genes that belong to clusters 2 to 8 and the members in each cluster are coded with the same colors as indicated above (**a**). **c**, In the classification with *k* = 8, clusters 1,2,3,4,6, and 8 are shown with the number of cluster members and selective modules representing specific GO terms. Clusters 5 and 7 are shown in Fig. 5d,e.

Supplementary Fig. 7

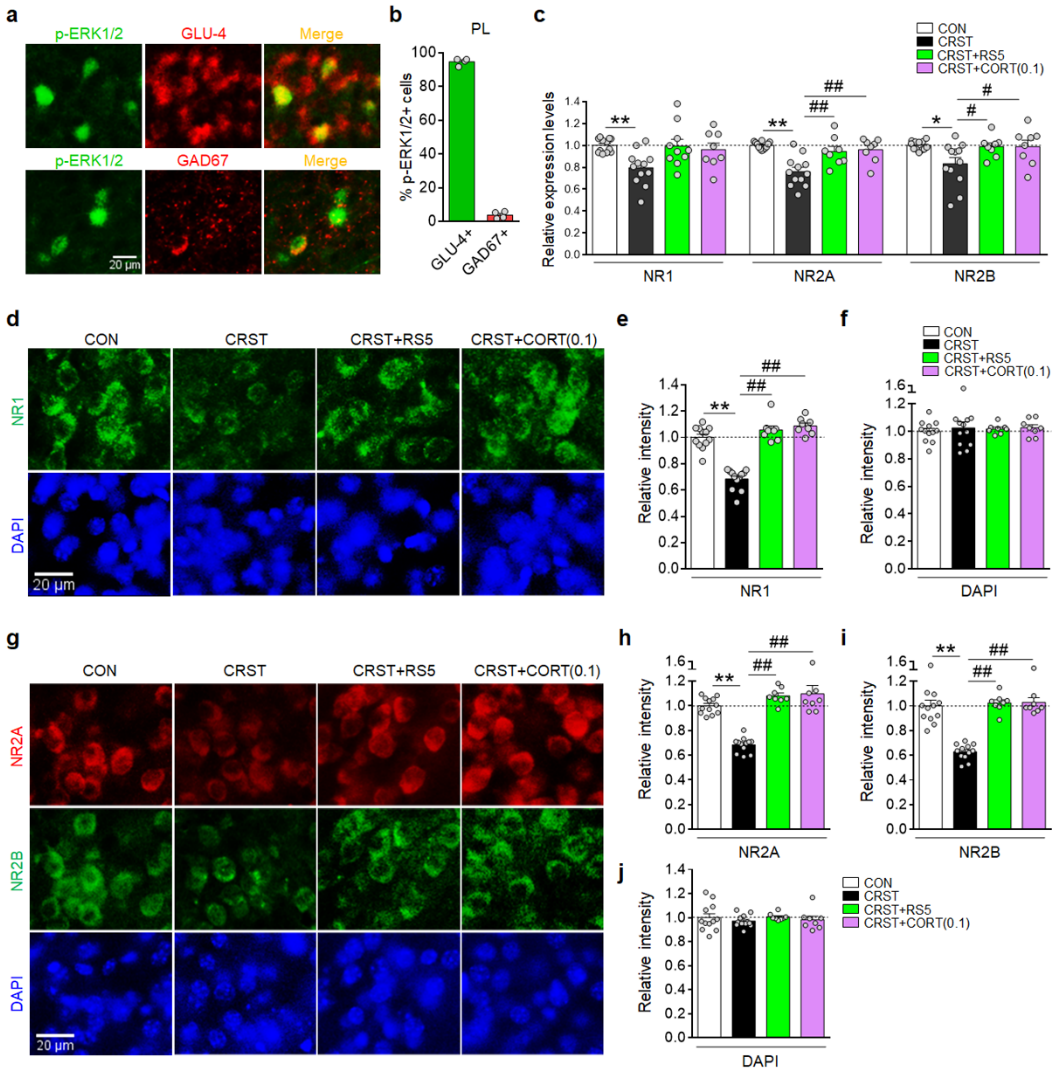

Supplementary Fig. 7 RS5 and low-dose CORT treatment in CRST mice upregulates the reduced expression of NMDAR subunits in the PL. a,**b**, Immunofluorescence staining of p-ERK1/2 (green) in PL neurons stained with GLU-4 (a marker of glutamate neurons; red) or GAD67 (a marker of GABA neurons, red) (**a**) and their quantification levels (**b**) (*n* = 4 per group). **c**, Transcript levels of the NMDA receptor subunits NR1, NR2A, and NR2B in the PL of the CON, CRST, CRST + RS5, and CRST + CORT (0.1 mg kg^{-1}) groups. Sample groups were prepared from the experiments depicted in Fig. 5g,h (*n* = 7–12 per group). **d**–**f**, Immunofluorescence staining of NR1 (green) expression in the PL (**d**) for the indicated groups, and quantification levels of NR1 (**e**) and DAPI intensity (**f**) (*n* = 4–6 animals per group). DAPI, blue. **g**–**j**, Immunofluorescence staining of NR2A (red) and NR2B (green) expression in the PL (**g**) for the indicated groups. DAPI, blue. Quantification levels of NR2A (**h**) and NR2B (**i**), and DAPI intensity (**j**) (*n* = 4–6 animals per group). Data are mean ± SEM. Gray circles represent individual data points. *, difference compared to control; #, difference compared to CRST. *, #, $p < 0.05$, **, ##, $p < 0.01$ (One-way ANOVA followed by Newman-Keuls post-hoc test). See Supplementary Data 4 for statistical details.

Supplementary Fig. 8

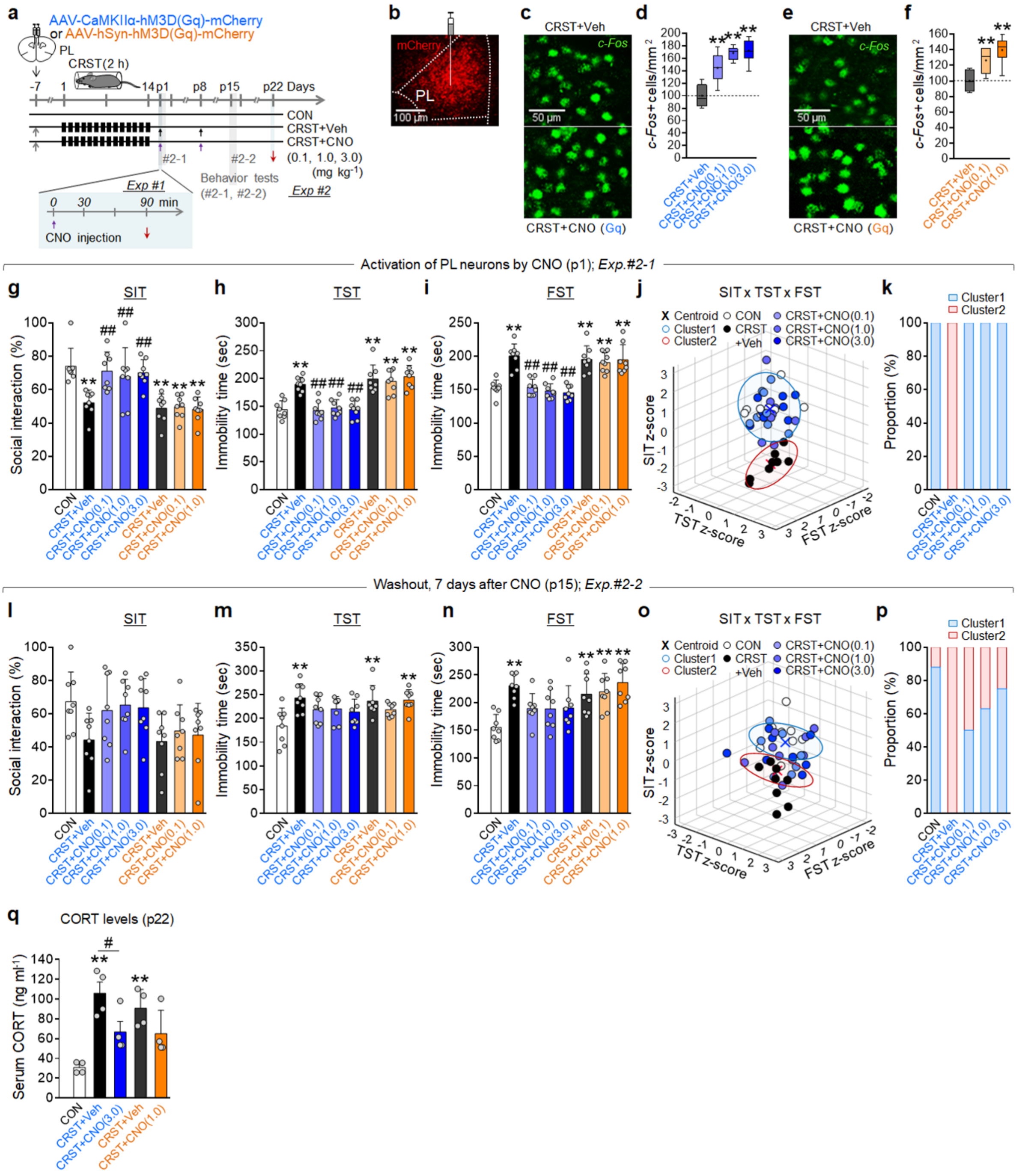

Supplementary Fig. 8 Chemogenetic activation of PL glutamatergic neurons in CRST mice improves stress-induced depressive behaviors. a–f, Experimental design (**a**). AAV8-CaMKIIα-hM3D(Gq)-mCherry excitatory vector was injected into the PL. Mice were then subjected to CRST. CNO or Veh injection, respectively, purple and black arrows. The expression of the hM3D(Gq)-mCherry fusion protein used here is under the control of a neuron-specific CaMKIIα promoter that ensures that mCherry-positive neurons are obligate to express hM3D(Gq). A photomicrograph showing mCherry expression by the injected vector in the PL (**b**). CNO-stimulated *c-Fos* induction in the PL in mice carrying AAV8-CaMKIIα-hM3D(Gq)-mCherry (**c** and **d**) or

AAV8-hSyn-hM3D(Gq)-mCherry (**e** and **f**) (Exp #1). CNO doses, 0.1, 1.0, or 3.0 mg kg^{-1}. **g**–**k**, The % time of social interaction in the SIT (**g**), and immobility time in the TST (**h**) and FST (**i**) for the indicated groups on post-stress day 1 (Exp #2-1). *K*-Means clustering of individuals in the SIT x TST x FST matrix (**j**) and the % composition of each group in the clusters (**k**) (*n* = 8 per group). **l**–**p**, The % time of social interaction in the SIT (**l**), and immobility time in the (**m**) and FST (**n**) for the indicated groups after CNO wash-out on post-stress day 15 (Exp #2-2). *K*-Means clustering of individuals in the SIT x TST x FST matrix (**o**) and the % composition of each group in the clusters (**p**) (*n* = 8 per group). **q**, Basal serum CORT levels in the indicated groups on post-stress day 22 (*n* = 8 per group). Data are mean ± SEM. Gray circles represent individual data points. *, difference compared to control; #, difference compared to CRST. *, #, $p < 0.05$, **, ##, $p < 0.01$ (One-way ANOVA followed by Newman-Keuls post-hoc test). See Supplementary Data 4 for statistical details.

Supplementary Fig. 9

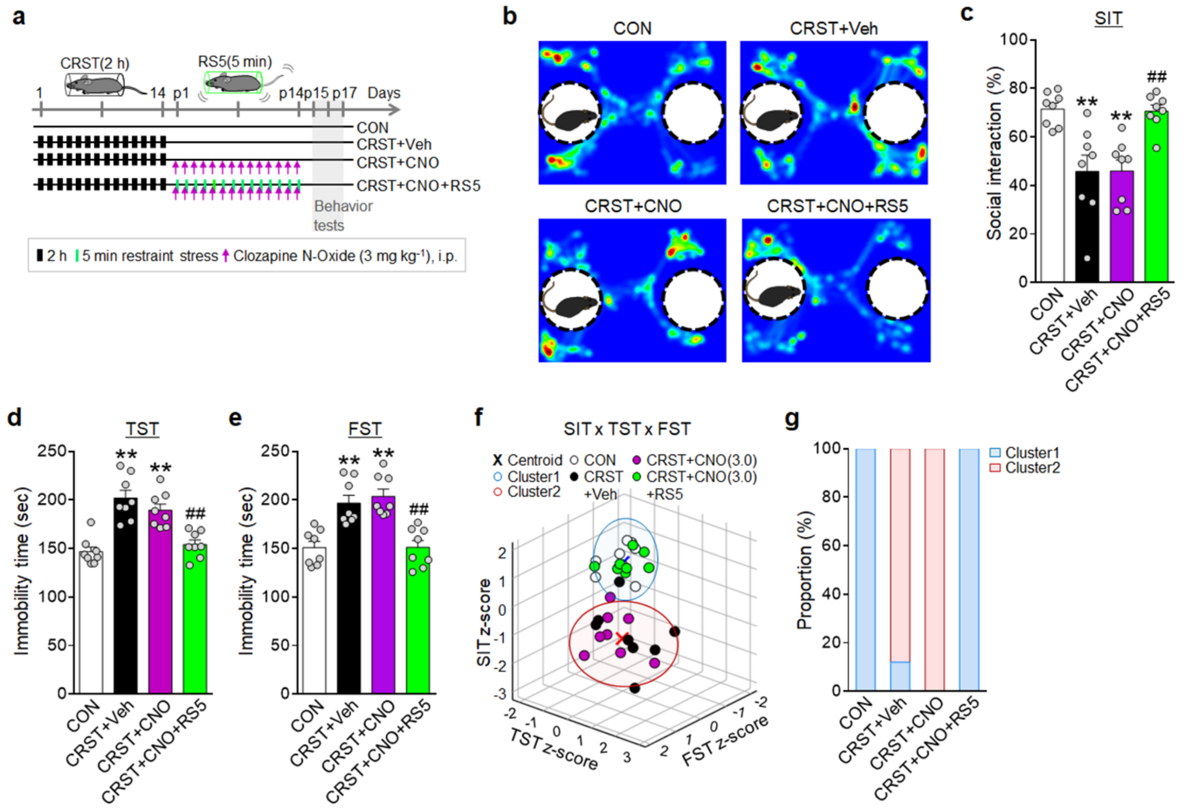

Supplementary Fig. 9 Repeated CNO treatment does not change depressive-like behaviors in CRST mice. a–g, Experimental design (**a**). Mice were exposed to CRST, followed by CNO injection (3 mg kg^{-1} per injection, i.p.) or RS5 with CNO injection for 14 days as depicted. Representative tracks (**b**) and the % time of social interaction in the SIT (**c**), and immobility time in the TST (**d**) and FST (**e**) for the indicated groups. *K*-Means clustering of individuals in the SIT x TST x FST matrix (**f**) and the % composition of each group in the clusters (**g**) ($n = 8$ per group). Data are mean ± SEM. Gray circles represent individual data points. **, difference compared to control; ##, difference compared to CRST. **, ##, $p < 0.01$ (One-way ANOVA followed by Newman-Keuls post-hoc test). See Supplementary Data 4 for statistical details.

Supplementary Fig. 10

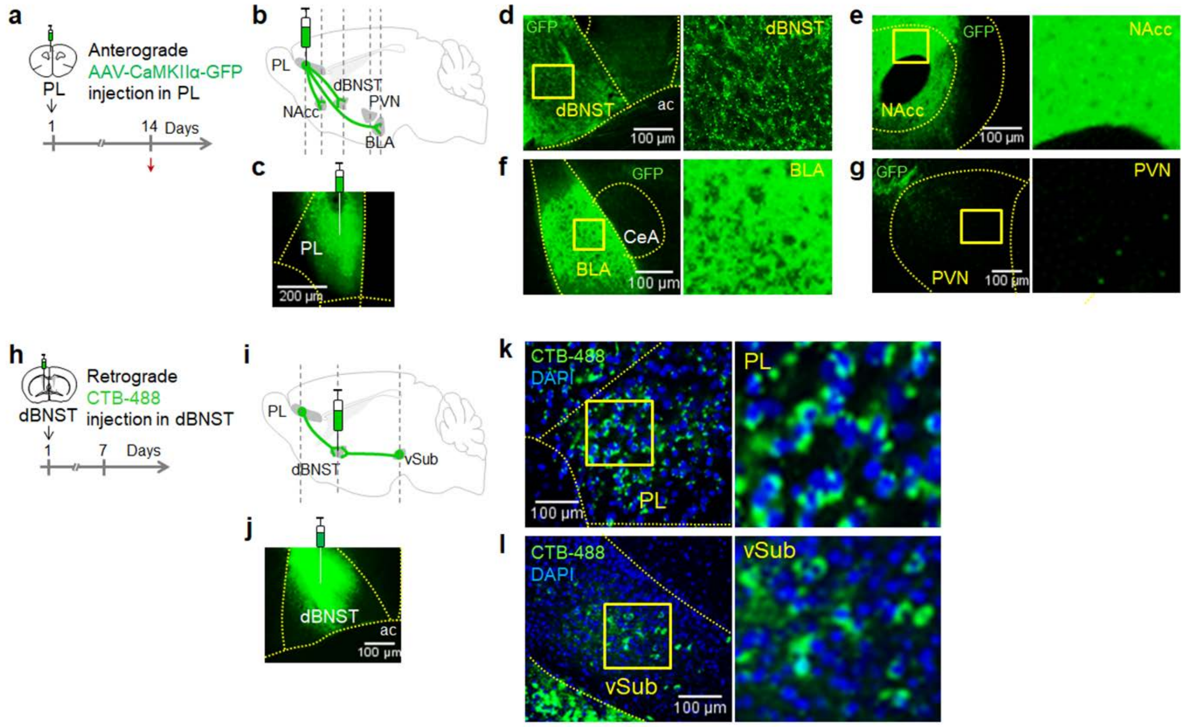

Supplementary Fig. 10 PL neurons projecting to the dBNST, NAcc and BLA are visualized by transducing with AAV-CaMKIIα-GFP expression. a–f, Experimental design (**a**). The anterograde tracer AAV-CaMKIIα-GFP was injected into the PL region (**b** and **c**), and two weeks later, subject mice were sacrificed and GFP expression in the brain was examined. Photomicrographs showing GFP signals in the dBNST (**d**), NAcc (**e**), BLA (**f**), and PVN (**g**) regions. High magnification of the area marked with a box for each region is indicated at the right side. dBNST, dorsal BNST, CeA, central amygdala; BLA, basolateral amygdala; NAcc, NAc core; PVN, paraventricular nucleus of the hypothalamus. **h–l**, Experimental design (**h**). The retrograde tracer CTB488 was injected into the dBNST (**i** and **j**). A week later, mice were sacrificed and CTB488 expression was analyzed. Photomicrographs showing CTB488 expression in the PL (**k**) and ventral subiculum (vSub) (**l**). High magnification of the area marked with a box for each region is indicated at the right side.

Supplementary Fig. 11

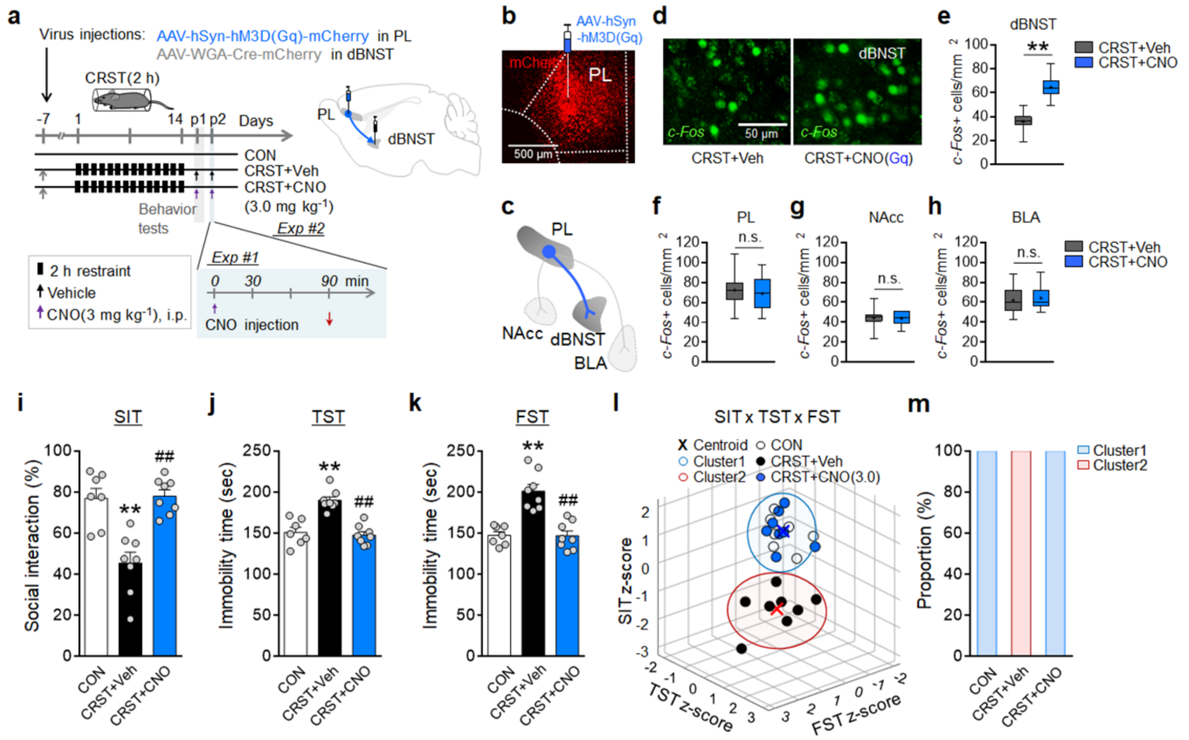

Supplementary Fig. 11 Chemogenetic activation of the PL→dBNST circuit rescues stress-induced depressive phenotypes of CRST-treated mice. a–h, Experimental design (**a**). AAV8-hSyn-hM3D(Gq)-mCherry was injected into the PL and a retrograde Cre vector was injected into the dBNST. Mice were then subjected to CRST. CNO or Veh was injected at indicated timepoints. A photomicrograph showing mCherry expression by the injected viral vector in the PL (**b**). A diagram showing the PL→dBNST circuit transduced with hM3D(Gq) expression. The dBNST, PL, NAcc, and BLA regions were examined for c-Fos expression (red boxes) (**c**). *c-Fos* expression levels in the dBNST (**d** and **e**), PL (**f**), NAcc (**g**) and BLA (**h**) in mice with the hM3D(Gq) expression in the PL→dBNST circuit after CON or Veh injection (Exp #1) (n = 6–9 per group). CNO dose, 3.0 mg kg^{-1} per injection (i.p.). Red arrow (↓) in Exp #1, sample prep point. **i–m**, The % time of social interaction in the SIT (**i**), and immobility time in the TST (**j**) and FST (**k**) for the indicated groups on post-stress day 1 (Exp #2). *K*-Means clustering of individuals in the SIT x TST x FST matrix (**l**) and the % composition of each group in the clusters (**m**) (n = 7–8 per group). Data are mean ± SEM. Gray circles represent individual data points. **, difference compared to control; ##, difference compared to CRST. **, ##, $p < 0.01$ (Two-sided Student's t-test; One-way ANOVA followed by Newman-Keuls post-hoc test). n.s., not significant. See Supplementary Data 4 for statistical details.

Supplementary Fig. 12

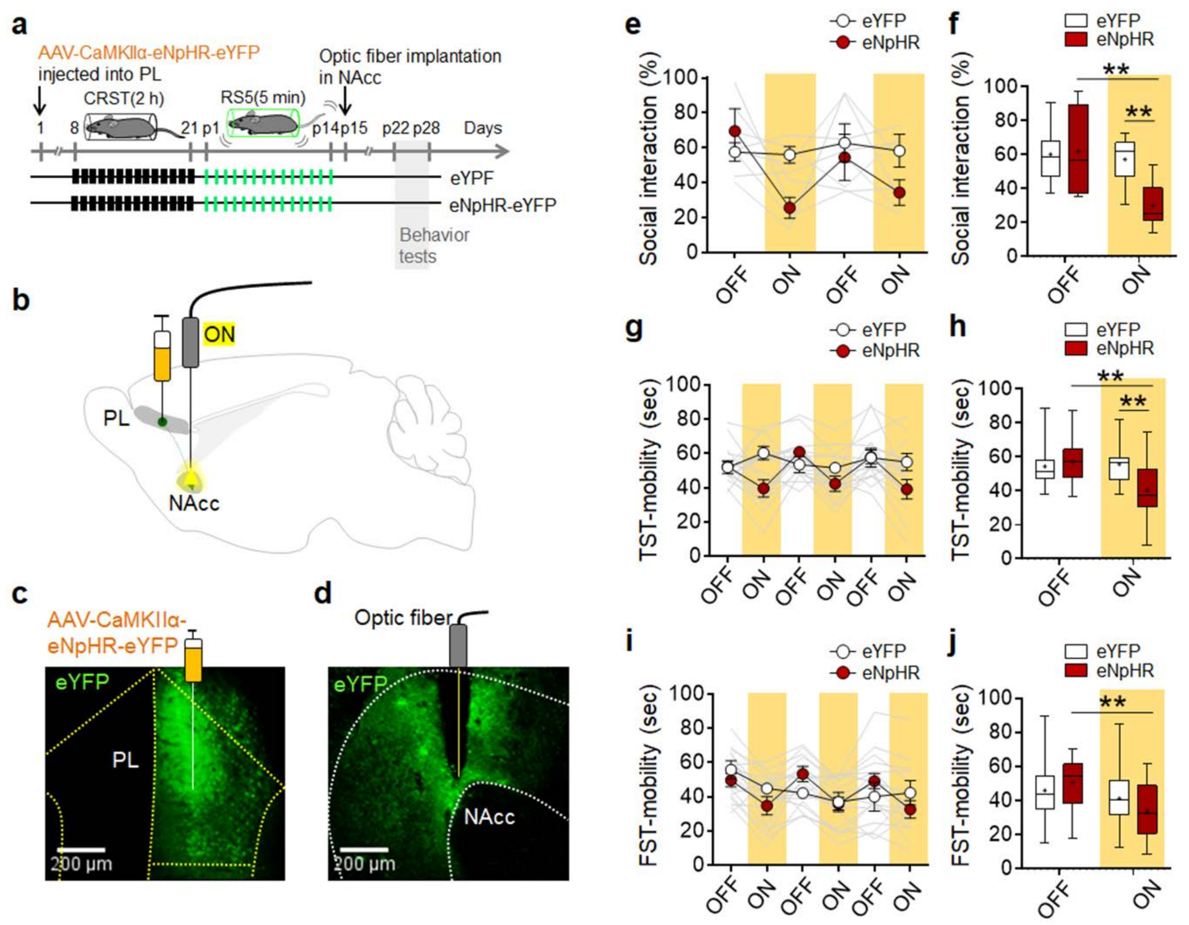

Supplementary Fig. 12 Optogenetic inhibition of PL neurons projecting to the NAcc produces depressive-like behaviors in RS5-treated CRST mice. a–d, Experimental design (**a**). AAV2/1-CaMKIIα-eNpHR-eYFP injected into the PL. Mice were then subjected to CRST, treated with RS5, and then received an optic fiber implantation into the NAcc. A diagram showing AAV2/1 vector injection into the PL and optic fiber implantation into the NAcc (**b**). Photomicrographs showing eNpHR-eYFP expression in PL (**c**) and NAcc (**d**) regions. **e–j**, Changes of social interaction (%) by ON-OFF stimulation (**e**) in the SIT and their summed data in the ON and OFF phases (**f**). Changes of mobility time (sec) by ON-OFF stimulation in the TST (**g**) and FST (**i**) and their summed data in the ON and OFF phases (**h, j**). Optic inhibition with two repeats of a 3-min ON-OFF session in the SIT (**e,f**) and three repeats of a 2-min ON-OFF session in the TST and FST (**g-j**) (ON session, yellow shade). n = 4–6 animals per group and two repeats. Data are mean ± SEM. Gray circles represent individual data points. **, difference compared to the indicated group. **, $p < 0.01$ (Two-sided Student's t-test). See Supplementary Data 4 for statistical details.
