# Supplementary material for: Repeated exposure with short-term behavioral stress resolves pre-existing stress-induced depressive-like behavior in mice
Source: Nat Commun. 2021 Nov 18;12:6682. doi: 10.1038/s41467-021-26968-4 (PMC8602389; doi:10.1038/s41467-021-26968-4)
Supplement: Supplementary file 3 — Description of Additional Supplementary Files [file 41467_2021_26968_MOESM3_ESM.pdf]

Description of Additional Supplementary Files

File name: Supplementary Data 1
Description: Supplementary Data 1. Short-term stress or low-dose glucocorticoid induces *c-Fos* expression differentially in various brain regions.

File name: Supplementary Data 2
Description: Supplementary Data 2. A list of the 264 genes that were upregulated or downregulated ≥1.2-fold in the PL after CRST treatment.

File name: Supplementary Data 3
Description: Supplementary Data 3. Supplementary Table 3. A list of the 722 genes that were upregulated or downregulated ≥1.2-fold in the PL after RS5 treatment in CRST mice.

File name: Supplementary Data 4
Description: Supplementary Data 4. Statistical Analysis

File name: Supplementary Data 5
Description: Supplementary Data 5. List of primer sequences used for RT-PCR analysis in this study
