# Supplementary material for: Repeated exposure with short-term behavioral stress resolves pre-existing stress-induced depressive-like behavior in mice
Source: Nat Commun. 2021 Nov 18;12:6682. doi: 10.1038/s41467-021-26968-4 (PMC8602389; doi:10.1038/s41467-021-26968-4)
Supplement: Supplementary file 9 — Reporting Summary [file 41467_2021_26968_MOESM9_ESM.pdf]

nature portfolio

Corresponding author(s): Pyung-Lim Han

Last updated by author(s): Sep 9, 2021

Reporting Summary

Nature Portfolio wishes to improve the reproducibility of the work that we publish. This form provides structure for consistency and transparency in reporting. For further information on Nature Portfolio policies, see our Editorial Policies and the Editorial Policy Checklist.

Statistics

For all statistical analyses, confirm that the following items are present in the figure legend, table legend, main text, or Methods section.

n/a	Confirmed	
☐	☒	The exact sample size (n) for each experimental group/condition, given as a discrete number and unit of measurement
☐	☒	A statement on whether measurements were taken from distinct samples or whether the same sample was measured repeatedly
☐	☒	The statistical test(s) used AND whether they are one- or two-sided<br>*Only common tests should be described solely by name; describe more complex techniques in the Methods section.*
☒	☐	A description of all covariates tested
☐	☒	A description of any assumptions or corrections, such as tests of normality and adjustment for multiple comparisons
☐	☒	A full description of the statistical parameters including central tendency (e.g. means) or other basic estimates (e.g. regression coefficient) AND variation (e.g. standard deviation) or associated estimates of uncertainty (e.g. confidence intervals)
☐	☒	For null hypothesis testing, the test statistic (e.g. F, t, r) with confidence intervals, effect sizes, degrees of freedom and P value noted<br>*Give P values as exact values whenever suitable.*
☒	☐	For Bayesian analysis, information on the choice of priors and Markov chain Monte Carlo settings
☒	☐	For hierarchical and complex designs, identification of the appropriate level for tests and full reporting of outcomes
☒	☐	Estimates of effect sizes (e.g. Cohen's d, Pearson's r), indicating how they were calculated

Our web collection on statistics for biologists contains articles on many of the points above.

Software and code

Policy information about availability of computer code

Data collection	The sociability test was carried out using a video tracking system (SMART; Panlab S.I., Barcelona, Spain) and collected data were analysed using SMART software (Version 3.0; Panlab S.I., Barcelona, Spain). Optical images were captured using MethMorph software (Molecular Devices, Sunnyvale, CA, USA). Microarray data were extracted using the Illumina GenomeStudio v2011.1 software (Gene Expression Module v1.9.0). Corticosterone absorbance was measured using SoftMax Pro software version 5.4. The quantitative real-time PCR (qPCR) data were obtained using Bio-Rad CFX Manager version 3.1 (Bio-rad, Hercules, CA, USA).
Data analysis	Immunostained images of brain sections were analyzed using a MetaMorph Microscopy Automation & Image Analysis software (Molecular Devices, Sunnyvale, CA, USA) or Image J analysis software version 1.51k (NIH Image, Bethesda, MD, USA). Western blot images were quantified using Image J analysis software version 1.51k (NIH Image, Bethesda, MD, USA). Gene expression profiles were analyzed using Multiexperiment Viewer (MeV), version 4.9. GO enrichment analysis was carried out using the STRING (Search Tool for Retrieval of Interacting Genes/Proteins) v11 database. Interactions in functional PPI networks were color-coded using Cytoscape StringApp v3.7.2. Corticosterone levels were analyzed using an online data analysis tool for ELISA of Enzo Life Science (http://www.myassays.com). Statistical analyses were conducted using GraphPad Prism 6 software (GraphPad Software, Inc., CA, USA). PCA and K-Means clustering were carried out using SPSS 25.0 software (IBM, Armonk, NY, USA). The Bio-Rad CFX Manager version 3.1 was used to analyze qPCR data.

For manuscripts utilizing custom algorithms or software that are central to the research but not yet described in published literature, software must be made available to editors and reviewers. We strongly encourage code deposition in a community repository (e.g. GitHub). See the Nature Portfolio guidelines for submitting code & software for further information.

Data

Policy information about availability of data

All manuscripts must include a data availability statement. This statement should provide the following information, where applicable:

- Accession codes, unique identifiers, or web links for publicly available datasets
- A description of any restrictions on data availability
- For clinical datasets or third party data, please ensure that the statement adheres to our policy

All data collected or analyzed in this study are available within the article and Supplementary Information. Further information and requests for resources and reagents should be directed to and will be fulfilled by the corresponding author. The gene expression microarray data generated in this study has been deposited in the NCBI Gene Expression Omnibus (GEO) under the accession number GSE183624. The transcriptomic datasets are also provided in Supplementary Tables 2 and 3. Source data are provided with this paper.

Field-specific reporting

Please select the one below that is the best fit for your research. If you are not sure, read the appropriate sections before making your selection.

[x] Life sciences [ ] Behavioural & social sciences [ ] Ecological, evolutionary & environmental sciences

For a reference copy of the document with all sections, see nature.com/documents/nr-reporting-summary-flat.pdf

Life sciences study design

All studies must disclose on these points even when the disclosure is negative.

Sample size	The sample sizes and other statistical details applied for all figures were indicated in Supplementary Table 4. Sample size determination is not based on power analysis.
Data exclusions	Mice were excluded from analysis when they showed bleeding or wounds owing to attacks by cage mates.
Replication	Key behavioral tests were repeated with multiple independent experiments.
Randomization	Animals were randomly assigned to control or experimental groups. All behavioral tests among groups and within groups were conducted in a randomized fashion or in an alternating manner with respect to test order and position within the testing equipment or the test field (e.g., left vs. right side, between positions in the test field)
Blinding	Data collection and analysis were not performed in a blind manner. However, all data were analyzed either by automated software or by examiners who were blind to the experimental conditions.

Reporting for specific materials, systems and methods

We require information from authors about some types of materials, experimental systems and methods used in many studies. Here, indicate whether each material, system or method listed is relevant to your study. If you are not sure if a list item applies to your research, read the appropriate section before selecting a response.

Materials & experimental systems

n/a	Involved in the study	
[ ]	[x]	Antibodies
[x]	[ ]	Eukaryotic cell lines
[x]	[ ]	Palaeontology and archaeology
[ ]	[x]	Animals and other organisms
[x]	[ ]	Human research participants
[x]	[ ]	Clinical data
[x]	[ ]	Dual use research of concern

Methods

n/a	Involved in the study	
[x]	[ ]	ChIP-seq
[x]	[ ]	Flow cytometry
[x]	[ ]	MRI-based neuroimaging

Antibodies

Antibodies used	Primary antibodies used in the present study are as follows:<br>- rabbit anti-c-Fos (sc-52, Santa Cruz Biotechnology, Lot#3016, 1:300 for IF, 1:2000 for IHC, RRID: AB_2106783)<br>- mouse anti-c-Fos (sc-271243, Santa Cruz Biotechnology, Lot#K1516, 1:300, RRID: AB_10610067)<br>- mouse anti-GLU-4 (G9282, Sigma-Aldrich, Lot#036M4871V, 1:300, RRID: AB_259989)<br>- mouse anti-GAD67 (MAB5406, Millipore, Lot#2775517, 1:250, RRID: AB_2278725)

March 2021

- mouse anti-GR (sc-136209, Santa Cruz Biotechnology, 1:500, Lot#A1818, RRID: AB_2251670)
- rabbit anti-GR (12041S, Cell Signaling Technology, Lot#3, 1:500, RRID: AB_2631286)
- mouse anti-Fkpb5 (GTX84491, Genetex, Lot#822000601, 1:50, RRID: AB_10728919)
- goat anti-NR1 (sc-1467, Santa Cruz Biotechnology, Lot#1508, 1:50, RRID: AB_670215)
- rabbit anti-NR2A (sc-9056, Santa Cruz Biotechnology, Lot#L0604, 1:50, RRID: AB_670228)
- mouse anti-NR2B (ab93610, Abcam, Lot#GR3257880-11, 1:100, RRID: AB_10561972)
- rabbit anti-pCaMKIIα (Thr286) (sc-12886-R, Santa Cruz Biotechnology, Lot#E2013, 1:2000 for WB, 1:1000 for IF, RRID: AB_2067915)
- rabbit anti-pERK1/2 (Thr202/Tyr204) (4370s, Cell Signaling Technology, Lot#24, 1:500 for WB and IHC, RRID: AB_2315112)
- mouse anti-CaMKIIα (A-1) (sc-13141, Santa Cruz Biotechnology, Lot#D1814, 1:2000 for WB, RRID: AB_626789)
- mouse anti-ERK1/2 (MK1) (sc-135900, Santa Cruz Biotechnology, Lot#A2315, 1:2000 for WB, RRID: AB_2141283)
- mouse anti-β-actin (C4) (sc-47778, Santa Cruz Biotechnology, Lot#1018, 1:3000 for WB, RRID: AB_2714189).

Secondary antibodies used in the present study are as follows:
- goat anti-rabbit IgG DyLight488 (DI-1488, Vector Laboratories, Lot#ZB1205, 1:500, RRID: AB_2336402)
- horse anti-rabbit IgG DyLight594 (DI-1094, Vector Laboratories, Lot#Z0905, 1:500, RRID: AB_2336414)
- horse anti-mouse IgG DyLight488 (DI-2488, Vector Laboratories, Lot#ZC0927, 1:500, RRID: AB_2307439)
- horse anti-mouse IgG DyLight594 (DI-2594, Vector Laboratories, Lot#ZG0127, 1:500, RRID: AB_2336412)
- mouse anti-goat IgG-FITC (sc-2356, Santa Cruz Biotechnology, Lot#B2217, 1:200, RRID: AB_628489)
- goat anti-rabbit IgG-HRP (sc-2004, Santa Cruz Biotechnology, 1:2000, RRID: AB_631746)
- goat anti-mouse IgG-HRP (sc-2005, Santa Cruz Biotechnology, Lot#H0415, 1:2000, RRID: AB_631736)
- goat anti-rabbit IgG (BA-1000, Vector Laboratories, Lot#ZB1007, 1:200, RRID: AB_2313606)
- goat anti-mouse IgG (BA-9200, Vector Laboratories, Lot#Y0907, 1:200, RRID: AB_2336171)

Validation

Antibodies used in the present study were commercially available and validated by the manufacturers or used in published research articles.
- rabbit anti-c-Fos (sc-52) “Validated in IHC, ICC/IF. Cited in 565 publication(s); reference article: PMID: 27539656; more relevant papers on https://www.scbt.com/p/c-fos-antibody-4”
- mouse anti-c-Fos (sc-271243) “Validated in IF. Cited in 24 publication(s); more relevant papers on https://www.scbt.com/p/c-fos-antibody-c-10”
- mouse anti-GLU-4 (G9282) “Validated in IF. Cited in 15 publication(s); reference article: PMID: 27539656; more relevant papers on https://www.sigmaaldrich.com/catalog/product/sigma/g9282”
- mouse anti-GAD67 (MAB5406) “Validated in IF in published reference; more relevant info on https://www.merckmillipore.com/product/Anti-GAD67-Antibody-clone-1G10.2,MM_NF-MAB5406”
- mouse anti-GR (sc-393232) “Validated in IF in published reference; more relevant info on https://www.scbt.com/p/gr-antibody-g-5”
- rabbit anti-GR (12041S) “Validated in IF in published reference; more relevant papers on https://www.cellsignal.com/products/primary-antibodies/12041”
- mouse anti-Fkpb5 (GTX84491) “Suitable for ICC/IF, IHC-P, WB; more relevant info on https://www.genetex.com/Product/Detail/FKBP5-antibody-3E9/GTX84491”
- goat anti-NR1 (sc-1467) “Validated in IF. Cited in 79 publication(s); Suitable for IF, IP, WB, ELISA, FC/FACS; more relevant info on https://datasheets.scbt.com/sc-1467.pdf”
- rabbit anti-NR2A (sc-9056) “Validated in IF. Cited in 17 publication(s); Suitable for IF, IHC, WB; more relevant info on https://www.scbt.com/p/nmdaepsilon1-antibody-h-54”
- mouse anti-NR2B (ab93610) “Validated in ICC/IF; Reference article: PMID: 31936514; more relevant info on https://www.abcam.com/nmdar2b-antibody-n5936-ab93610.html”
- rabbit anti-pCaMKIIα (Thr286) (sc-12886-R) “Validated in IF and WB. Cited in 18 publication(s); reference article: PMID: 25863961, PMID: 31267372; more relevant info on https://www.scbt.com/ko/p/p-camkiialpha-antibody-thr-286”
- rabbit anti-pERK1/2 (Thr202/Tyr204) (4370s) “Validated in IF and WB. Cited in 3796 publication(s); reference article: PMID: 25863961, PMID: 31267372; more relevant info on https://www.cellsignal.com/products/primary-antibodies/4370”
- mouse anti-CaMKIIα (A-1) (sc-13141) “Suitable for WB; reference articles: PMID: 25863961 and PMID: 31267372; more relevant info on https://www.scbt.com/p/camkiialpha-antibody-a-1”
- mouse anti-ERK1/2 (MK1) (sc-135900) “Suitable for WB; reference articles: PMID: 25863961 and PMID: 31267372; more relevant info on https://www.scbt.com/p/erk-1-2-antibody-mk1”
- mouse anti-β-actin (C4) (sc-47778) “Suitable for WB; reference article: PMID: 31267372; more relevant info on https://www.scbt.com/p/beta-actin-antibody-c4”
- Secondary antibodies (DI-1488, DI-1094, DI-2488, DI-2594, sc-2004, sc-2005, BA-1000 and BA-9200) were suitable for IF, IHC and WB. Reference articles: PMID: 25863961 and PMID: 31267372.

Animals and other organisms

Policy information about studies involving animals; ARRIVE guidelines recommended for reporting animal research

Laboratory animals

Seven-week-old male C57/BL6 mice were purchased from Daehan BioLink (Eumsung, Chungbuk, Republic of Korea). Eight-week-old male and female ICR (CD1) mice were purchased from OrientBio. Inc. (Seongnam, Gyeonggi, Republic of Korea) and used as breeders. All mice were housed in pairs in standard clear plastic cages in a temperature (23–24°C)- and humidity (50–60%)-controlled room, with food and drinking water available ad libitum. The animal room was maintained on a 12-hour light/dark cycle (light on at 7 AM) in a specific-pathogen-free environment.

Wild animals

This study does not involve wild animals.

Field-collected samples

This study does not involve field-collected samples.

Ethics oversight	Animal experiments were performed in accordance with the animal care guidelines of Ewha Womans University, and all protocols used in the present study were approved by the Institutional Animal Care and Use Committee (IACUC16-018 and IACUC19-015).

Note that full information on the approval of the study protocol must also be provided in the manuscript.
